# Rootstock effects on bitter pit incidence in ‘Honeycrisp’ apples are associated with changes in fruit’s cell wall chemical properties

**DOI:** 10.3389/fpls.2022.1034664

**Published:** 2022-10-13

**Authors:** Md Tabibul Islam, Jianyang Liu, Protiva Rani Das, Amritpal Singh, Sherif M. Sherif

**Affiliations:** ^1^ Alson H. Smith Jr. Agricultural Research and Extension Center, School of Plant and Environmental Sciences, Virginia Tech, Winchester, VA, United States; ^2^ Summerland Research and Development Centre, Science and Technology Branch, Agriculture and Agri-Food Canada, Summerland, BC, Canada

**Keywords:** Honeycrisp, rootstock, Ca^2+^, pectin, bitter pit (BP)

## Abstract

Bitter pit (BP) is a physiological disorder of apples that often appears during or after cold storage. Despite being defined as a calcium deficiency disorder, BP is a complex process that is not only affected by the total Ca^2+^ content in the fruit but also by the proper cellular Ca^2+^ homeostasis and partitioning. Early investigations have also suggested that rootstocks could affect BP development and severity. In the present study, rootstock effects on BP development were assessed on ‘Honeycrisp’ trees that were grafted on 14 different rootstocks (B.10, G.11, G.202, G.214, G.30, G.41, G.935, G.969, M.26 EMLA, M.9, V.1, V.5, V.6, and V.7). We evaluated BP incidence at harvest, and three months after cold storage for four, and three growing seasons, respectively. BP incidence was significantly reduced in ‘Honeycrisp’ trees on B.10 compared to other rootstocks, whereas trees on V.6 showed the highest percentage of BP at harvest and after cold storage. ‘Honeycrisp’ apples were collected from three different rootstocks (B.10, G.41, and V.6) two months after cold storage and evaluated for mineral nutrient composition, Ca^2+^ homeostasis, and cell wall properties, e.g., pectin content, pectin de-esterification rate and pectin methylesterase (PME) activity. Water-soluble and insoluble pectin content was markedly higher in fruits from B.10 than in G.41 and V.6. We also observed increased PME enzyme activity and a greater degree of water-insoluble pectin de-esterification in ‘Honeycrisp’ apples from V.6 compared to those from B.10. A significantly higher Ca^2+^ was found in the fruits from B.10 than G.41 and V.6. Higher Ca^2+^ and lower Mg^2+^ levels were also observed in the cell wall and water-insoluble pectin fractions of the fruits from B.10 compared to G.41 and V.6. However, the ratio of cell wall-bound Ca^2+^ to total Ca^2+^ was lower in B.10 compared to G.41 and V.6. Together, our results indicate that the tolerance of B.10 to BP could be attributed to a reduced PME activity and lower pectin de-esterification level, which in turn reduced the amount of Ca^2+^ cross-linked with pectin, and probably increased the apoplastic free calcium concentrations that is essential for maintaining cell membrane integrity and reducing BP development.

## Introduction

‘Honeycrisp’ is the third most-produced apple cultivar in the USA, after ‘Gala’ and ‘Red Delicious’ (Donahue et al., 2021). The high market value and consumer demand for this variety is largely due to its unique fruit quality characteristics (juicy, crispy, and strong-flavored) and palatability at harvest, which are sustained during storage (Al Shoffe et al., 2020). However, several production and storage difficulties, including bitter pit (BP) disorder, are associated with ‘Honeycrisp’. It is estimated that growers lose on average 20% of the ‘Honeycrisp’ crop due to BP per year, which could reach up to 80% in severe cases, causing significant economic losses to the entire apple industry in the United States (Cheng and Sazo, 2018).

BP is a physiological disorder of apples that often appears during or after cold storage but can also emerge pre-harvest. BP manifests itself symptomatically as dark, sunken lesions on the peel, and brown, oxidized flesh that can extend up to 1 cm below the fruit surface. BP has been defined as a Ca^2+^ deficiency disorder in fruit tissue (de Freitas et al., 2010); however, BP incidence is not always linked to total Ca^2+^ content in the fruit, suggesting a more complicated cause for BP (Falchi et al., 2017). Indeed, several studies have indicated that BP disorder may involve abnormal cellular Ca^2+^ homeostasis. For instance, an increase of Ca^2+^ storage in the vacuole and/or binding to the cell wall can lead to a reduction of the free apoplastic Ca^2+^, which could explain the finding that the pitted fruits may have similar or even higher total Ca^2+^ levels compared to healthy fruits (Chamel and Bossy, 1981; Saure, 2005; de Freitas et al., 2010). Other nutrients that have also been linked to BP development in apples include magnesium (Mg^2+^), potassium (K^+^), and nitrogen (N). High Mg^2+^ and K^+^ levels were observed in fruit tissues with Ca^2+^ deficiency disorders (de Freitas et al., 2010). Mg^2+^ competes with Ca^2+^ for binding sites in the cell wall and may replace Ca^2+^ but cannot pursue its functions in maintaining cell wall integrity (Yermiyahu et al., 1994). Similarly, the high levels of N can lead to Ca^2+^ deficiency by promoting vegetative growth and subsequently the movement of Ca^2+^ ions in the transpiration stream to leaves instead of fruits (Lim and White, 2005). Therefore, it has been widely accepted that the ratios of K^+^/Ca^2+^, N/Ca^2+^, and Mg^2+^/Ca^2+^ are better indicators of BP incidence than the total Ca^2+^ content (Dris et al., 1998; Lanauskas and Kvikliene, 2006; de Freitas et al., 2010; Fazio et al., 2020; Marini et al., 2020).

Ca^2+^ is predominantly bound to the fruit’s cell wall, specifically with pectin, to maintain its integrity (White and Broadley, 2003; de Freitas et al., 2010). Pectins are an essential class of polysaccharides present in the cell wall and composed mainly of galacturonic acid. The presence of ester and ionic bonds between adjacent pectins contributes to the physical properties of fruit cell walls (de Freitas et al., 2010; Hocking et al., 2016). The activity of different pectin metabolizing enzymes can also affect the structural complexity of pectins. For instance, previous study showed that alterations in the expression of genes related to pectin degradation and modification, such as *polygalacturonase*, *pectate lyase*, and *pectin methylesterase* (PME) can affect Ca^2+^ homeostasis, its binding capacity to the cell wall, and the content of free Ca^2+^ available for normal cellular metabolism (de Freitas et al., 2010). De-esterification of pectin by PME also increases the number of carboxyl groups that can form electrovalent bonds with Ca^2+^ ions in the cell wall and reduces the levels of apoplastic-free Ca^2+^, eventually leading to BP incidence (Goulao and Oliveira, 2008; de Freitas et al., 2010). PME enzyme is also involved in cell wall disassembly during fruit ripening (Ralet et al., 2001).

The occurrence of BP is, by and large, a disbalance of mineral composition in fruits, which is subject to many factors, such as soil conditions, the vegetative to reproductive growth ratio, tree age, crop load and rootstocks. In addition to its effects on several horticultural traits, apple rootstocks have a diverse influence on the nutritional status of the tree canopy and could also be implicated in the physiology and occurrence of BP. Rootstock’s influence on BP was explained in light of rootstock differences in the absorption and transport of certain nutrients, which in turn affect the composition of the scion’s levels of K^+^, Mg^2+^ and Ca^2+^. For instance, in a study conducted by Donahue et al., 2021, the authors studied the effects of three rootstocks, M.9, M.26 and B.9 on BP incidence in ‘Honeycrisp’ apples and revealed that the peel Mg^2+^/Ca^2+^ ratio and peel Ca^2+^ level could be a good indicator of BP incidence. In a separate study, using a more genetically-diverse group of apple rootstocks, Fazio et al., 2020, showed that rootstocks have a significant impact on the Ca^2+^, K^+^, and K^+^/Ca^2+^ ratio in the scion tissues, and that K^+^/Ca^2+^ in the leaves and fruit of the scion was tightly correlated with BP incidence. However, rootstock influences on the fruit’s cell-wall remodeling of and mineral nutrient portioning in relation to BP development, particularly during cold storage, remained unexplored. The present study aimed to characterize i) the effects of 14 different rootstock on BP development in ‘Honeycrisp’ apples, ii) the correlation between different fruit quality parameters and BP development, and iii) the impact of rootstocks on fruit’s cell wall chemical properties during cold storage. It is worth noting that the effects of the same set of rootstocks on tree growth parameters and yield was previously published as a part of a multi-location study (Cline et al., 2021) and in an extension publication (Sherif, 2022).

## Materials and methods

### Plant materials, location, and experimental design

In this study, ‘Honeycrisp’ trees on 14 different rootstocks, including Budagovsky.10 (B.10); Geneva rootstocks, G.11, G.202, G.214, G.30, G.41, G.935, G.969; Malling rootstocks, M.26 EMLA, M.9-T337; and Vineland rootstocks, V.1, V.5, V.6, V.7; were planted at the Saunders Brothers Orchard, Piney River, Virginia, United States (39°06′36.0 ′N 78°16′48.0 ′W) in the spring of 2014 as a part of a multi-state program, NC-140, that is supported by the United States Department of Agriculture- Specialty Crop Research Initiative (USDA-SCRI). All the trees were trained to a tall spindle training system and spaced at 1.22 m within the row and 3.66 m between rows (2240 trees ha^-1^). The irrigation, fertilization, pest control, and disease management for this block followed local guidelines. The trees were arranged in two rows according to the completely randomized design (CRD), with ten single-tree replicates per each rootstock. To prevent biennial bearing, the crop load of each tree was managed by hand thinning, leaving a single fruit per cluster, and no more than 5-6 fruit/cm^2^ of trunk cross-sectional areas (TCSA). Trees were defruited in 2014 and allowed to fruit in 2016. Details about trial location, soil type and irrigation were previously published (Cline et al., 2021). Fertilization, pest and disease management were carried out following the local spray bulletin guidelines (https://www.pubs.ext.vt.edu/456/456-419/456-419.html).

### Bitter pit incidence and fruits quality assessment

Bitter pit (BP) (%) was determined using 25-50 fruits per tree depending on the crop volume. Fruits were evaluated for BP (%) at harvest for four seasons (2018-2021) and after three months of cold storage for three seasons (2018, 2020-2021). A fruit with one pit was recorded as a pitted fruit based on the visible symptoms. The BP (%) of each rootstock was presented as the average of 5 trees (seasons 2018 & 2019) or 10 trees (seasons 2020 & 2021).

Fruit quality was assessed immediately after harvest. At each assessment, 5-10 apples were used from each tree to measure fruit diameter, weight, firmness and total soluble solids (TSS) according to methods described previously by (Allen et al., 2021). Fruit firmness was recorded for the sun and shade side of each fruit using a Fruit Texture Analyzer (FTA, QA Supplies, Norfolk, VA). Soluble solids content (SSC) was determined using a digital refractometer (Atago 3810, Japan).

### Fruit tissue sampling for analyzing cell wall properties

‘Honeycrisp’ apples from trees grafted on three different rootstocks (B.10, G.41, and V.6) were collected after two months of storage at 4°C to measure the pectin content, pectin de-esterification level, pectin methyl esterase (PME) enzyme activity, and Ca^2+^ and Mg^2+^ concentration. Fruit skin and flesh samples were taken from the calyx-end of 10 fruit per replication in the 2021 growing season. The skin tissues with about 1 mm thickness were obtained by peeling the fruit with a potato peeler. The flesh tissues with about 3 mm thickness were collected from the outer portion of the calyx region with a knife. All samples were frozen in liquid N_2_, ground to a fine power using a Geno/Grinder (SPEXSamplePrep, Metuchen, NJ, USA) and stored at −80°C until further analysis.

### Cell wall preparations

The ground skin and flesh tissues were used for all cell wall extractions following a method described previously by de Souza et al., 2014, with minor modification. Briefly, 3 g of fresh tissues were homogenized in 95% ethanol (1.5 L kg^−1^, fresh weight) for 20 min, then centrifuged at 4000 × g for 10 min. After centrifugation, the supernatant was decanted and the pellet was successively washed with 1:1 (v/v) chloroform: methanol, washed again with acetone and then air-dried. This dried pellet was considered as the alcohol insoluble cell wall substance (AIS). Samples of the AIS were suspended in distilled H_2_O for one hour and centrifuged (10,000 × g) for 15 minutes. The supernatant was collected, and the previous step was repeated once. The two supernatants were combined and designated as the water-soluble pectin fraction, whereas the pellets that remained after centrifugation was considered to contain the water-insoluble pectin fraction.

### Mineral nutrients analysis

Fruit skin tissues collected as described previously by Marini et al., 2020 was dried at 70°C for 48 hours prior to analyzing the Ca^2+^, Mg^2+^, and K^+^ concentration, and the samples were subjected to microwave acid digestion/dissolution and then analyzed by inductively coupled plasma atomic emission spectrometry (Meyer and Keliher, 1992) and considered as total Ca^2+^, Mg^2+^, and K^+^ content. The Ca^2+^ and Mg^2+^ concentrations were also measured in the cell wall substances and the water-insoluble pectin fraction of both skin and flesh tissues. The Ca^2+^ and Mg^2+^ concentrations in the water-soluble pectin were obtained by subtracting their respective content of water-insoluble fraction from that found in the cell wall substance. The soil nutrient content was analyzed according to a previously described method (Gavlak et al., 2005) and presented in the **Supplementary Table S1**. The mineral nutrient content in the leaves from trees on B.10, G.41, and V.6 was also analyzed (**Supplementary Table S2**) following the method described previously (Gavlak et al., 2005).

### Total uronic acid quantification

The water-soluble pectin fraction was dried using vacuum centrifugations and dried fractions were dissolved with 67% sulfuric acid and used for the uronic acid quantification. The total uronic acid content of each pectin fraction was measured based on the method described by van den Hoogen et al. (1998). Briefly, 40 µl sample was taken in each well of a 96-wells microtiter plate, and 200 µl concentrated sulfuric acid [96% (w/w)] containing 120 mM sodium tetraborate was carefully added. After mixing the sample and the reagent, the plate was placed in an incubator for one hour at 80°C. After cooling to ambient temperature, the background absorbance of the samples was measured at OD 540 nm on a Synergy H1 hybrid reader (BioTek, Oakville, ON, Canada). Then 40 µl of *m*-hydroxydiphenyl reagent [100 ml of *m*-hydroxydiphenyl in dimethyl sulfoxide, 100 mg/ml, mixed with 4.9 ml 80% (v/v) sulfuric acid just before use] was added and mixed with the samples. After 15 min, the absorbance of the pink-colored samples was reread at OD 540 nm. The background absorbance was subtracted from the second reading, and the uronic acid content was obtained using a linear calibration curve of galacturonic acid.

### Pectin’s de-esterification rate

The degree of de-esterification was measured by the reductive method in which the esterified galacturonosyl carboxyl groups were reduced (i.e., converted from galacturonic acid to galactose) and then the de-esterification uronic acids were quantified and represented as a percentage of the total uronic acid content present in the original unreduced samples (Klein et al., 1995). Duplicate samples of water-soluble and water-insoluble pectins were weighed into 2 ml eppendorf tube, and one sample was incubated overnight in 1 ml of 10 mg m1^-1^ NaBH_4_ in 50% EtOH. This sample was then neutralized with acetic acid (HOAc), dried, and washed several times with HOAc: methanol (MeOH) (1:9) and then once with MeOH. Both duplicates were then dissolved in 67% H_2_SO_4_, and the uronic acid content was determined as described previously. Incubation with NaBH_4_ converts esterified uronosyl residues to galactose, so the difference in uronic acid content between the duplicate samples was the amount of uronic acid which contained methyl esters.

### Pectin methyl esterase activity

The crude protein was extracted from apple skin and flesh tissues using 100 mM Tris-HCl buffer (pH 7.5) and used for the PME enzyme activity analyses according to the methods described by Liu et al. (2013) with some modifications. Briefly, 100 mg ground frozen samples were suspended in 100 mM Tris-HCl. The supernatants were collected for enzyme assay after centrifugation at 12,000 g for 15 min. The reaction mixture (180 µl) contained 156 µl of 0.4 mM NAD, 20 µl of 0.5% (w/v) pectin (from citrus peel, P9135, Sigma), 2 µl of 0.35 U formaldehyde dehydrogenase (from Pseudomonas putida, F1879, Sigma), and 2 µl of 0.1 U alcohol oxidase (from P. pastoris, A2404, Sigma). The reaction was initiated by the addition of 10 µl crude protein and the conversion of the NAD^+^ to NADH was recorded at OD 340 nm over 15 min using a microplate reader (Synergy H1 hybrid reader, BioTek, Oakville, ON, Canada). The change in absorption per unit time over the linear part of the reaction was calculated for each well and used to calculate the increase in the concentration of NADH. The NADH concentration was calculated using extinction coefficient Ɛ340 for NADH (6,220 M^-1^cm^-1^). One-unit (U) PME activity is defined as one µmol NADH formed per min.

### Statistical analysis

The data was compiled using Microsoft Excel software version 2016 (Microsoft, Redmond, Washington, USA). JMP 16.2.0 software (SAS Institute Inc, Carey, NC) was used for one-way ANOVA and Pearson’s correlation analysis. Tukey’s HSD was used to compare means whenever the model was significant based on ANOVA. The means were compared at α = 0.05 significance level. The statistical analyses of the biochemicals and the nutrient data were conducted using the software SAS 9.1.3 (SAS Institute Inc., Cary, NC, United States). Tukey’s HSD test was employed to compare the means of separate replicates. Statistical significance was postulated at *P* < 0.05.

## Results

### The effect of rootstocks on bitter pit incidence

**Figure 1:**
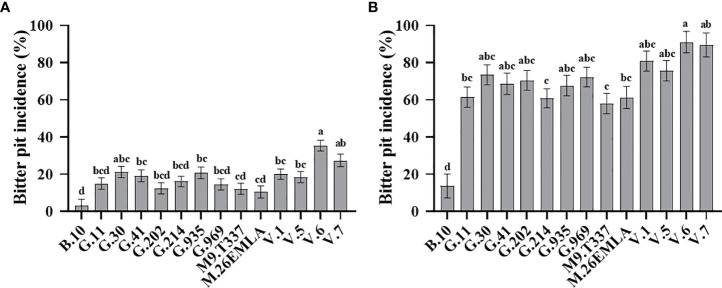
Rootstock effects on bitter pit (BP) incidence in ‘Honeycrisp’ apples. **(A)** Cumulative BP incidence at harvest (each bar represents the average of four growing seasons); **(B)** cumulative BP incidence after three months of cold storage (each bar represents the average of three growing seasons). Means not sharing any letter are significantly different at α = 0.05., according to Tukey’s HSD test in JMP Pro 16.

Rootstock significantly affected BP (%) in ‘Honeycrisp’ apples at harvest and after cold storage (**Figure 1**). Our 4-year evaluation of fruits at harvest showed that ‘Honeycrisp’ trees on B.10 had the lowest BP incidence, whereas those on V.6 had the highest BP incidence (**Figure 1A**). A moderate level of BP was observed in trees grafted on G.11, G.41, G.202, G.214, G.935, G.969, M.26-EMLA, M.9-T337, V.1, and V.5 (**Figure 1A**). The BP (%) followed the same trend for fruits analyzed after three months of cold storage, with those on B.10 showing the least and those on V. series showing the highest BP incidence. Again, a moderate level of BP was observed in trees grafted on G.214, G.11, M.9-T337, and M.26 EMLA (**Figure 1B**).

### Rootstock effects on fruit quality and the relationship between fruit quality and bitter pit incidence

**Figure 2:**
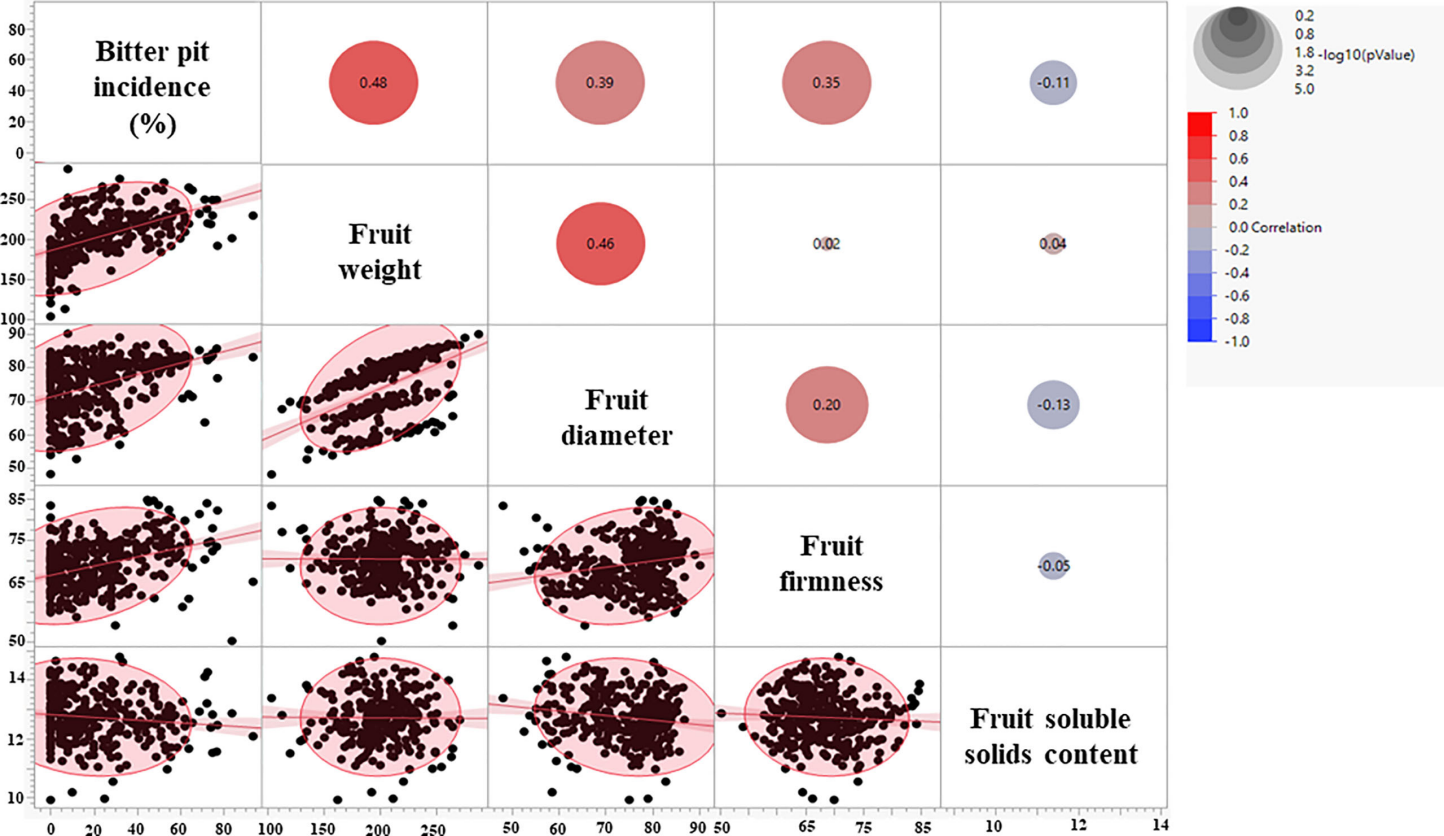
Correlation coefficients between bitter pit incidence and fruit quality parameters at harvest.

Fruit quality parameters, including fruit weight, diameter, and firmness, were analyzed at harvest for four growing seasons (2018-2021), whereas soluble solids content (SSC) was evaluated for three growing seasons (2019-2021) (**Table 1**). The effects of rootstocks on fruit weight were significant, with ‘Honeycrisp’ apples on B.10 being the lightest (168 g), whereas those on V.5 and V.6 were the heaviest (220 gm). On the other hand, no significant differences in fruit diameter, firmness, and SSC were observed among different rootstocks (**Table 1**). Fruit weight, diameter, and firmness showed a significant positive correlation with BP incidence at harvest (**Figure 2**). The highest correlation value (r = 0.48) was recorded between fruit weight and BP incidence.

**Table 1:** Effect of different rootstocks on fruit weight, diameter, firmness, and soluble solids content of ‘Honeycrisp’ apples.

Rootstock	Fruit weight (g)	Fruit diameter (mm)	Fruit firmness (N)	Soluble solids content (Brix)
**B.10**	168 ± 6^c^	71 ± 2^a^	71 ± 1^a^	12.7 ± 0.2^a^
**G.11**	189 ± 6^bc^	74 ± 2^a^	71 ± 1^a^	12.7 ± 0.2^a^
**G.30**	215 ± 6^ab^	76 ± 2^a^	69 ± 1^a^	12.5 ± 0.2^a^
**G.41**	201 ± 6^ab^	75 ± 2^a^	71 ± 1^a^	12.8 ± 0.2^a^
**G.202**	191 ± 5^bc^	74 ± 1^a^	72 ± 1^a^	13.0 ± 0.2^a^
**G.214**	213 ± 5^ab^	76 ± 1^a^	70 ± 1^a^	12.7 ± 0.2^a^
**G.935**	210 ± 6^ab^	76 ± 2^a^	70 ± 1^a^	12.6 ± 0.2^a^
**G.969**	216 ± 6^ab^	76 ± 1^a^	70 ± 1^a^	12.5 ± 0.2^a^
**M.9 T337**	197 ± 6^ab^	74 ± 1^a^	70 ± 1^a^	12.9 ± 0.2^a^
**M.26 EMLA**	199 ± 6^ab^	75 ± 2^a^	71 ± 1^a^	12.8 ± 0.2^a^
**V.1**	203 ± 5^ab^	75 ± 1^a^	69 ± 1^a^	12.4 ± 0.2^a^
**V.5**	220 ± 6^a^	77 ± 2^a^	70 ± 1^a^	12.8 ± 0.2^a^
**V.6**	220 ± 5^a^	78 ± 2^a^	71 ± 1^a^	12.7 ± 0.2^a^
**V.7**	214 ± 6^ab^	77 ± 2^a^	71 ± 1^a^	13.0 ± 0.2^a^

Data represent the mean ± standard error, and different lowercase letters in a column indicate significant differences at P < 0.05 according to Tukey’s HSD test. Fruit weight, diameter, and firmness were presented as the average of four growing seasons (2018-2021), and soluble solids were presented as the average of three growing seasons (2019-2021).

### The concentration of Ca, Mg and K in ‘Honeycrisp’ apples is affected by the rootstock

**Figure 3:**
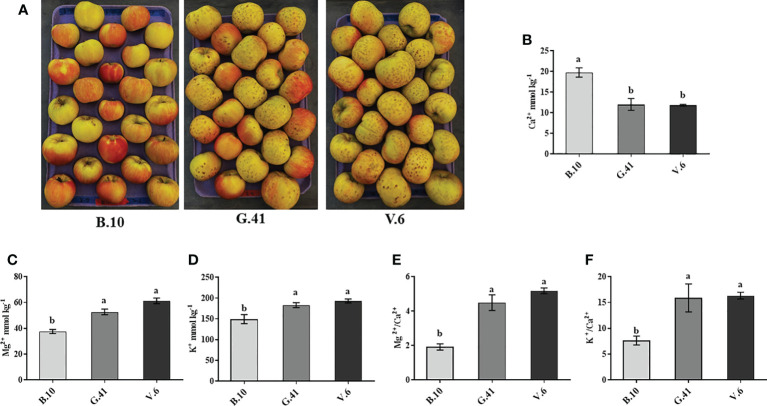
Variations in mineral nutrient concentrations in ‘Honeycrisp’ apples as affected by the rootstock. **(A)** Bitter pit (BP) symptom development on ‘Honeycrisp’ apples from B.10, G.41 and V.6 rootstocks after two months of cold storage. **(B–D)** concentration of calcium (Ca), magnesium (Mg) and potassium (K) and **(E, F)**, Mg/Ca and K/Ca ratios in ‘Honeycrisp’ apples’s skin tissues. Means not sharing any letter are significantly different at *P* < 0.05, according to Tukey’s HSD test.

After two months in cold storage, ‘Honeycrisp’ apples from three different rootstocks (B.10, G.41 and V.6) were analyzed for mineral nutrient concentration in fruit skin. Fruits on V.6 and G.41 showed more severe BP incidences compared to B.10 (**Figure 3A**). The total Ca^2+^ concentration in the skin tissue was significantly higher (164% and 167%) for fruits on B.10 compared to those on G.41 and V.6, respectively (**Figure 3B**). In contrast, Mg^2+^ and K^+^ concentrations were higher in the fruits from G.41 and V.6 than those from B.10 (**Figures 3C, D**). Similarly, the ratios of Mg^2+^/Ca^2+^ and K^+^/Ca^2+^ were significantly higher in fruits from G.41 and V.6 compared to B.10 (**Figures 3E, F**).

### Content and de-esterification rate of uronic acids

**Figure 4:**
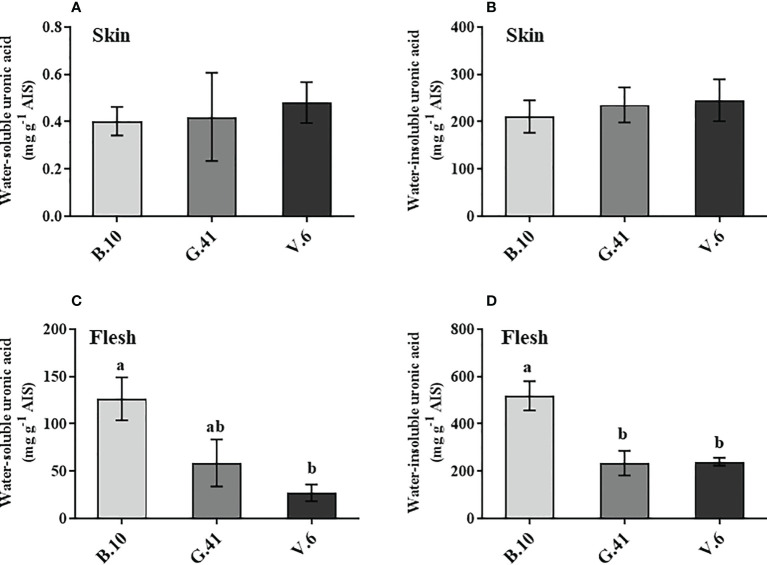
The uronic acid content in skin and flesh tissue of ‘Honeycrisp’ apples from different rootstocks. **(A, B)** Water-soluble and water-insoluble uronic acid in skin tissue, **(C, D)** Water-soluble and water-insoluble uronic acid in flesh tissues of ‘Honeycrisp’ apples from B.10, G.41 and V.6 after two months of cold storage. Means not sharing any letter are significantly different at *P* < 0.05, according to Tukey’s HSD test. AIS: Alcohol insoluble cell wall substance.

Similar to mineral nutrients, apples from B.10, G.41 and V.6 were collected two months after cold storage uronic acids quantification in the skin and flesh tissues. Total uronic acids were primarily present in the water-insoluble fractions in the skin tissue (**Figures 4A, B**) and neither water-soluble nor water-insoluble uronic acids showed significant differences among the rootstocks (**Figures 4A, B**). However, in the flesh tissues, the two forms of uronic acid showed significant differences among rootstocks (**Figures 4C, D**). Apples from the trees grafted on B.10 contained significantly higher amounts of uronic acid than those from G.41 and V.6, especially in the water-insoluble fraction of the flesh tissue (**Figure 4D**).

**Figure 5:**
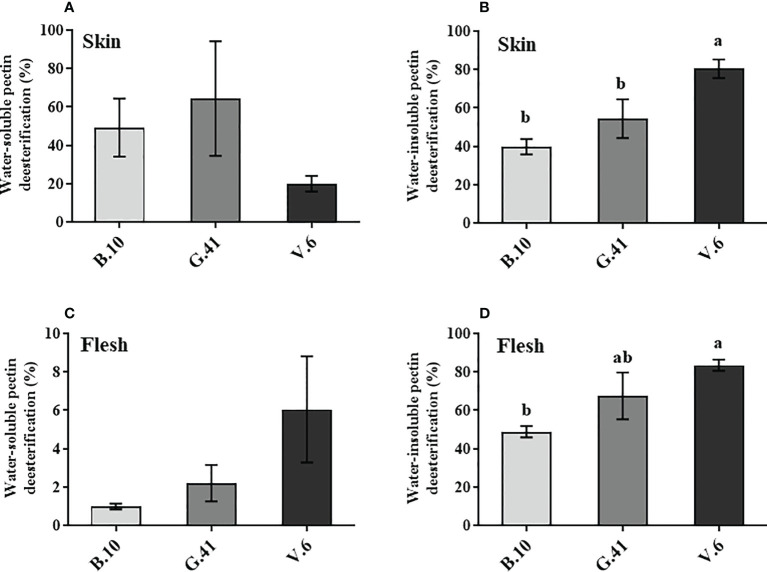
Effects of rootstocks on the de-esterification rate (%) of pectin in ‘Honeycrisp’ apples. **(A, B)** de-esterification rate (%) of water-soluble and water-insoluble pectin in skin tissues, **(C, D)** de-esterification rate (%) of water-soluble and water-insoluble pectin in flesh tissues of ‘Honeycrisp’ apples from B.10, G.41 and V.6 after two months of cold storage. Means not sharing any letter are significantly different at *P* < 0.05, according to Tukey’s HSD test.

The de-esterification rate of the water-soluble pectin in the skin and flesh tissues was not significant among rootstocks (**Figures 5A, C**). However, a significant difference was found in the de-esterification rate (%) of the water-insoluble pectin in both skin and flesh tissues, with fruits from B.10 showing a lower de-esterification rate than those from G.41 and V.6 rootstocks (**Figures 5B, D**).

### Pectin methylesterase enzyme activity

**Figure 6:**
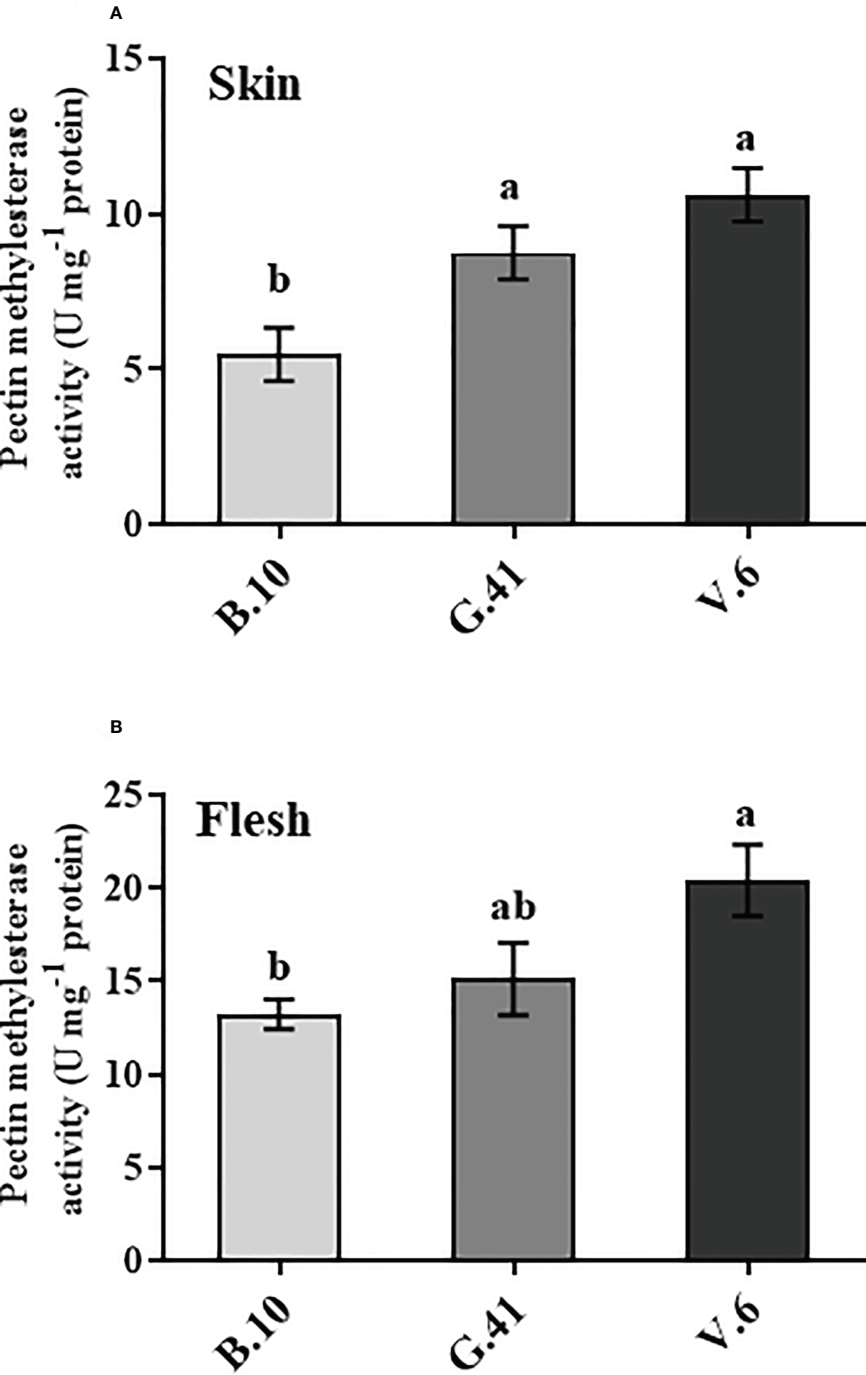
Pectin methyl esterase (PME) enzyme activity in ‘Honeycrisp’ apples from different rootstocks. The PME activity in the skin **(A)** and flesh **(B)** tissues of apples from B.10, G.41, and V.6 after two months of cold storage. Means not sharing any letter are significantly different at *P* < 0.05, according to Tukey’s HSD test.

The pectin methylesterase (PME) is the enzyme that catalyzes the de-esterification of the pectin. The fruits on V.6 showed significantly higher PME enzyme activity than B.10 in both skin and flesh tissues (**Figure 6**). The PME activity in the skin tissue was 160% and 194% higher in G.41 and V.6, respectively, compared to B.10.

### Rootstock effects of Ca^2+^ and Mg^2+^ partitioning in the cell wall

**Figure 7:**
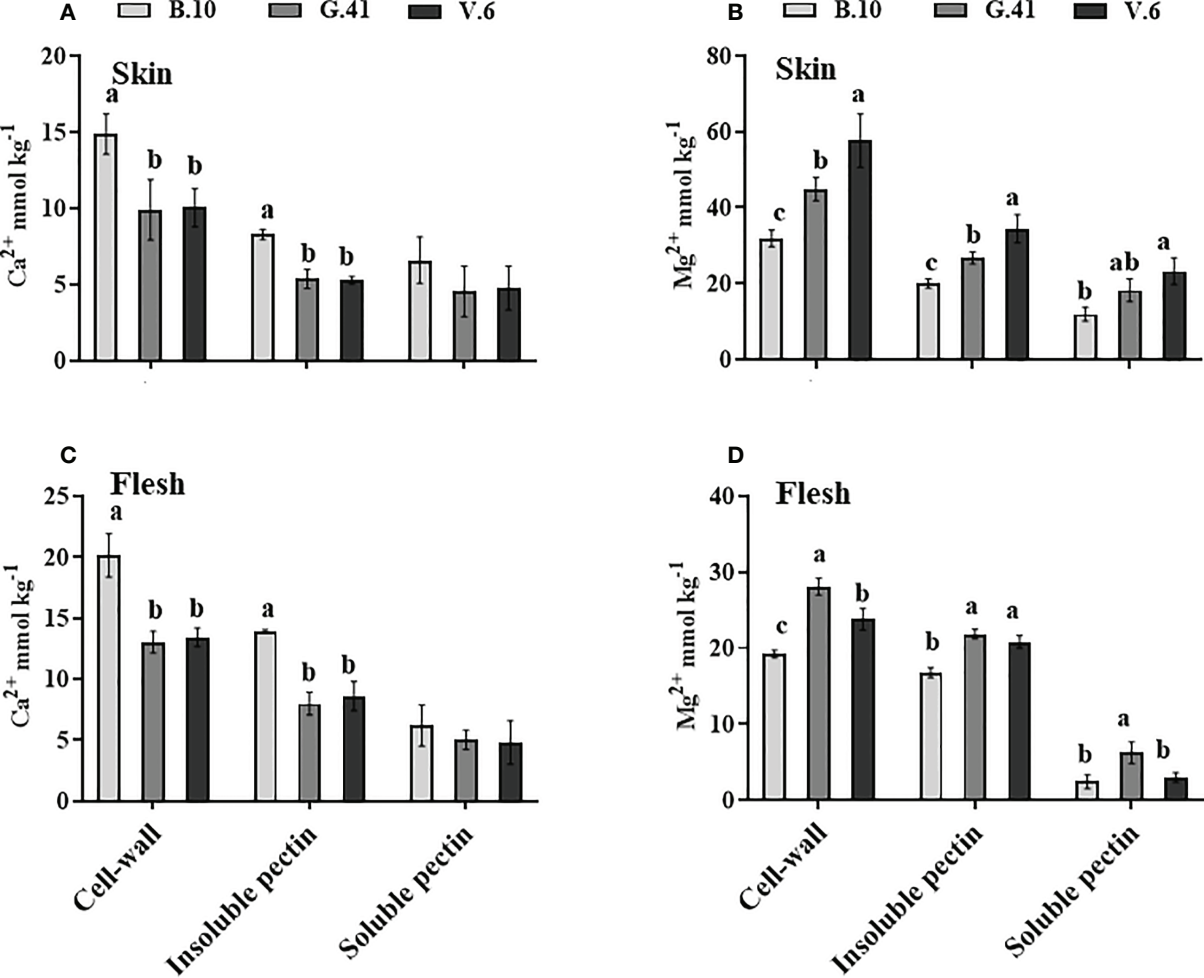
Rootstock effects on the Ca^2+^and Mg^2+^partitioning in the cell-wall materials of skin and flesh tissues of ‘Honeycrisp’ apples. **(A, C)** Ca^2+^ concentration in cell-wall, insoluble pectin, and soluble pectin of apples’ skin and flesh tissues, **(B, D)** Mg^2+^ concentration in cell-wall, insoluble pectin, and soluble pectin of apples’ skin and flesh tissues. Means not sharing any letter are significantly different at *P* < 0.05, according to Tukey’s HSD test.

Ca^2+^ concentration was significantly higher in the cell-wall and water-insoluble pectin fraction of apples from B.10 compared to G.41 and V.6 (**Figures 7A, C**) in both skin and flesh tissues. Conversely, Mg^2+^ concentration in the cell wall and water-insoluble pectin was significantly lower in the skin and flesh tissues of apples from B.10 compared to G.41 and V.6 (**Figures 7B, D**). A significant difference also existed between G.41 and V.6 for the Mg^2+^ concentration in the cell wall and water-insoluble pectin fraction of skin tissue (**Figure 7B**).

## Discussion

‘Honeycrisp’ apples are highly prone to bitter pit (BP), a physiological disorder that occurs on the apple peel and beneath. Different factors affect BP development in ‘Honeycrisp’, including rootstocks (Fazio et al., 2020; Donahue et al., 2021; Valverdi and Kalcsits, 2021). The choice of the rootstock is critical for several other horticultural performances, such as tree growth, yield, stress tolerance, fruit quality, adaptability to environmental conditions, etc. Previously, the rootstock effects on BP development in ‘Honeycrisp’ apples have been reported with a limited number of rootstocks. For instance, Donahue et al. (2021) evaluated the rootstock effects on BP incidence by using three different rootstocks (B.9, M.9, and M.26) and concluded that Budagovsky 9 (B.9) shows the lowest BP incidence compared to others. In the present study, we used 14 different rootstock genotypes to characterize the rootstock effects on BP development at harvest and three months after cold storage. Among the investigated rootstocks, B.10 showed the lowest, whereas V.6 and V.7 showed the highest BP incidence at harvest and after cold storage. All the Geneva^®^ series rootstocks (G.11, G.202, G.214, G.30, G.41, G.935, G.969) and M.26. EMLA and M.9-T337 also showed high BP incidences, especially after cold storage (**Figure 1**). The rootstocks examined in this trial were developed by different North American and European breeding programs. The Malling rootstocks, generally referred to as M., e.g., M.26, were developed at the East Malling Research Station, Kent, England. The Geneva series of rootstocks, referred to as G., e.g., G.41, were developed by the Geneva Apple Rootstock Breeding Program, a joint venture between Cornell University and the United States Department of Agriculture (USDA). Budagovsky rootstocks, referred to as Bud or B. (e.g., B.9 and B.10), were developed in the Soviet Union by crossing M.8 x Red Standard (Krasnij Standart). Vineland series of rootstocks, referred to as V. (e.g., V.1 and V.6), were developed at the Horticultural Experiment Station at Vineland, Ontario, from open-pollinated hybrids of ‘Kerr’ crabapples and M.9 rootstock (Cline et al., 2021; Sherif, 2022.) B.10 rootstock used in this study was developed from a B.9 X B.13-14 cross, and therefore its low BP incidence could be attributed to inheritance from B.9, a rootstock that is widely known for its relative tolerance to BP (Lordan et al., 2019; Donahue et al., 2021; Valverdi and Kalcsits, 2021). The increased tolerance of B.10 to BP could also be related to its influence on fruit weight and diameter. Among all rootstocks, B.10 produced the lightest ‘Honeycrisp’ apples and our study showed a positive correlation between fruit weight and diameter on the one hand and BP incidence on the other hand (**Figure 2**). The positive linear relationship between fruit size and BP in ‘Honeycrisp’ apples has also been previously reported (Ferguson and Watkins, 1989; Reid and Kalcsits, 2021; Marini et al., 2022).

BP is believed to be caused by Ca^2+^ deficiency in fruits. A negative correlation was observed between Ca^2+^ content and BP development in apple skin (Torres et al., 2017; Al Shoffe et al., 2020; Donahue et al., 2021). Similarly, in ‘Granny Smith’, the skin tissue of the non-BP apples showed significantly higher Ca^2+^ and lower Mg^2+^ and K^+^ contents compared to BP fruits after two months of cold storage (de Freitas et al., 2015). We also observed higher Ca^2+^ and lower Mg^2+^ and K^+^ in the skin tissues of the fruits from B.10 than G.41 and V.6, which could explain, at least partially, the more tolerance of B.10 to BP. Additionally, we found higher Ca^2+^ levels in the leaves of the trees on B.10 than G.41 and V.6 (**Supplementary Table S2**), revealing altogether the differential Ca^2+^ uptake capacity among rootstocks. It was also suggested that rootstocks affect BP development by regulating the mineral nutrient composition of fruits (Donahue et al., 2021). However, a discrepancy in rootstock influences on fruit’s content of mineral nutrients, particularly Ca^2+^, and its association with BP also existed. For instance, Valverdi and Kalcsits (2021) reported that ‘Honeycrisp’ fruit’s content of Ca^2+^ was not significantly different among the investigated rootstocks (B.9, G.41, G.890 and M.9) in two consecutive seasons and did not correlate with BP development. Such discrepancy might be due to the effect of rootstocks on Ca^2+^ partitioning into the cell, rather than total Ca^2+^ content. In fact, previous investigations reported that BP-damaged tissues have more Ca^2+^ than the surrounding healthy tissues (Val et al., 2008; de Freitas et al., 2010). Together, these observations led us to investigate the effects of rootstocks on ‘Honeycrisp’ fruit’s cell wall remodeling, particularly the effects on pectin levels, pectin de-esterification rate and Ca^2+^ concentration in cell wall materials.

The fruit cell wall is a dynamic structure and responds to developmental and environmental stimuli through structural remodeling, including modification and solubilization of pectin, calcium-pectin crosslinks formation, etc. (Hocking et al., 2016). The cell wall’s outer part, or ‘middle lamella,’ is shared by neighboring cells. The middle lamella is primarily made of polygalacturonic acid, also known as pectic acid or pectin. Ca^2+^ is involved in the linkage of the pectic substances of the cell wall by its electrostatic interaction with the carboxylic group of the pectin. The presence of Ca^2+^ increases cohesion and maintains cell wall integrity (Hocking et al., 2016), therefore the majority of cellular calcium (60%-75%) is bound to the cell wall (White and Broadley, 2003). In this study, the calyx-end tissues, where the frequency of BP incidence is often higher (Ferguson and Watkins, 1989) were analyzed for water-soluble and water-insoluble uronic acid content, and we found significantly lower levels of these two forms of pectin in the flesh tissue of apples from G.41 and V.6, compared to ‘B.10’. However, no significant changes in the skin tissue’s uronic acid content were observed among the rootstocks (**Figures 4A, B**), indicating that the degradation of pectin in flesh tissue underneath the skin might be associated with the higher BP in G.41 and V.6.

The ability of pectin to regulate adhesion and maintain cell wall integrity is determined not only by its levels, but also by chemical modifications, e.g. de-esterification, that affect its ability to gel. Pectin de-esterification is catalyzed by pectin methyl esterase (PMEs). This enzyme removes the methyl groups, exposing the carboxylic groups of pectin that can be crosslinked by Ca^2+^ and form calcium pectate (Hocking et al., 2016). Previous studies revealed that de-esterification of the cell wall pectin is closely related to the BP incidence during cold storage (de Freitas et al., 2010; de Freitas et al., 2015). The higher de-esterification rate has been found in the water-insoluble and water-soluble pectin of ‘Granny Smith’-BP fruits, concomitant with enhanced *PMEs* expression in the skin and flesh tissues in these fruits (de Freitas et al., 2010; de Freitas et al., 2015). In agreement with that, we observed a higher de-esterification rate in the water-insoluble pectin of the fruits on G.41 and V.6 compared to B.10 (**Figure 5**), which was also combined with enhanced PME enzyme activity (**Figure 6**). Similarly, tomatoes with silenced *PME* showed a lower de-esterification rate of the pectin than the wild type, especially in the water-insoluble pectin (de Freitas et al., 2012). Therefore, it could be postulated that the higher PME activity and the resulting de-esterification of water-insoluble pectin in ‘Honeycrisp’ apples from G.41 and V.6 could have created a Ca^2+^ deficient environment, leading to BP development. Also, at high concentrations, Ca^2+^ can work as a competitive inhibitor of the PME enzyme and decrease its affinity to bind with the substrate (Charnay et al., 1992). So, it can also be implied that more Ca^2+^ uptake by B.9 rootstocks could have affected the PME enzymatic activity and pectin de-esterification in ‘Honeycrisp’ apples, leading to less Ca^2+^ binding to the cell wall and more Ca^2+^ availability in the apoplast.

The Ca^2+^ and Mg^2+^ concentrations showed a contrasting pattern in the cell wall and the water-insoluble pectin fraction. Higher Ca^2+^ and lower Mg^2+^ levels were found in the fruits from B.10 compared to G.41 and V.6. This suggests that G.41 and V.6 may have been compensating for the Ca^2+^ shortage with Mg^2+^. Although Mg^2+^ can also interact with pectin to form magnesium pectate, it cannot replace Ca^2+^ function in the cell. For instance, Ca^2+^ binding to the phospholipids and proteins of the cell membrane is crucial for delaying lipid catabolism and preserving membrane integrity (Picchioni et al., 1996; Picchioni et al., 1998). Therefore, it could be suggested that the allocation of much Ca^2+^ to cell wall could reduce the apoplastic pool of free Ca^2+^, which could in turn affect cell membrane integrity leading to BP development. Indeed, we found a higher portion of Ca^2+^ (84% and 82%, respectively) bound to the cell wall of the fruits from V.6 and G.41 than B.10 (75%) (**Supplementary Table S3**).

In conclusion, this study demonstrates that rootstocks significantly affect BP incidence in ‘Honeycrisp’ apples. We found that trees on B.10 show less BP (%) at harvest and after three months of cold storage compared to the 13 other rootstocks investigated in this study. The biochemical analyses of apples from different rootstocks indicated that rootstocks not only affect fruit’s Ca^2+^, Mg^2+^, and K^+^ content but can also modulate cell wall structure and Ca^2+^ hemostasis by affecting pectin desertification rate and PME activity in fruit’s skin and flesh tissues. Our data also suggest that sensitivity to BP could be retributed to high PME enzyme activity and increased pectin de-esterification rate, which creates more Ca^2+^ binding sites in the cell wall, and less Ca^2+^ availability in the apoplast leading eventually to cell membrane breakdown and BP development.

## Data availability statement

The original contributions presented in the study are included in the article/**Supplementary Material**. Further inquiries can be directed to the corresponding author.

## Author contributions

MI contributed to conceptualization, investigation, methodology, formal analysis, validation, writing–original draft preparation, visualization. JL and PD methodology, formal analysis, review and editing. AS methodology, statistical analysis. SS conceptualization, validation, resources, data curation, writing–review and editing, supervision, project administration, funding acquisition. All authors have read and agreed to the published version of the manuscript.

## Funding

This project was partially funded by the Virginia Apple Research Program (# 467404) and USDA-NIFA (# VA-160095, and VA-136332)

## Acknowledgments

The authors thank Ms. Sara Pitcock, Ms. Mariah Temkin, Mr. Chester Allen, Mr. Kenneth Savia, for their assistance in orchard assessments and data collection and Mr. Noah Jacobs for his assistance in sample preparation.

## Conflict of interest

The authors declare that the research was conducted in the absence of any commercial or financial relationships that could be construed as a potential conflict of interest.

## Publisher’s note

All claims expressed in this article are solely those of the authors and do not necessarily represent those of their affiliated organizations, or those of the publisher, the editors and the reviewers. Any product that may be evaluated in this article, or claim that may be made by its manufacturer, is not guaranteed or endorsed by the publisher.

## References

[B1] Allen W. C. Kon T. Sherif S. M. (2021). Evaluation of blossom thinning spray timing strategies in apple. Horticulturae 7, 308. doi: 10.3390/horticulturae7090308

[B2] Al Shoffe Y. Nock J. F. Baugher T. A. Marini R. P. Watkins C. B. (2020). Bitter pit and soft scald development during storage of unconditioned and conditioned ‘Honeycrisp’ apples in relation to mineral contents and harvest indices. Postharvest Biol. Technol. 160, 111044. doi: 10.1016/j.postharvbio.2019.111044

[B3] Chamel A. R. Bossy J. P. (1981). Electron-microprobe analysis of apple fruit tissues affected with bitter pit. Sci. Hortic. 15, 155–163. doi: 10.1016/0304-4238(81)90103-5

[B4] Charnay D. Nari J. Noat G. (1992). Regulation of plant cell-wall pectin methyl esterase by polyamines “Interactions with the effects of metal ions. Eur. J. Biochem. 205 (2), 711–714. doi: 10.1111/j.1432-1033.1992.tb16833.x 1572369

[B5] Cheng L. Sazo M. (2018). Adjusting soil pH for optimum nutrient availability. Fruit Quarterly. 26 (1), 19–23.

[B6] Cline J. A. Autio W. Clements J. Cowgill W. Crassweller R. Einhorn T.. (2021). Early performance of ‘Honeycrisp’ apple trees on several size-controlling rootstocks in the 2014 NC-140 rootstock trial. J. Am. Pomol. 75, 189–202.

[B7] de Freitas S. T. Amarante C. V. T. Labavitch J. M. Mitcham E. J. (2010). Cellular approach to understand bitter pit development in apple fruit. Postharvest Biol. Technol. 57, 6–13. doi: 10.1016/j.postharvbio.2010.02.006

[B8] de Freitas S. T. do Amarante C. V. T. Mitcham E. J. (2015). Mechanisms regulating apple cultivar susceptibility to bitter pit. Sci. Hortic. 186, 54–60. doi: 10.1016/j.scienta.2015.01.039

[B9] de Freitas S. T. Handa A. K. Wu Q. Park S. Mitcham E. J. (2012). Role of pectin methylesterases in cellular calcium distribution and blossom-end rot development in tomato fruit. Plant J. 71 (5), 824–835. doi: 10.1111/j.1365-313X.2012.05034.x 22563738

[B10] de Souza A. Hull P. A. Gille S. Pauly M. (2014). Identification and functional characterization of the distinct plant pectin esterases PAE8 and PAE9 and their deletion mutants. Planta 240 (5), 1123–1138. doi: 10.1007/s00425-014-2139-6 25115560 PMC4200376

[B11] Donahue D. J. Reig Córdoba G. Elone S. E. Wallis A. E. Basedow M. R. (2021). ‘Honeycrisp’ bitter pit response to rootstock and region under Eastern new York climatic conditions. Plants 10 (5), 983. doi: 10.3390/plants10050983 34069071 PMC8155886

[B12] Dris R. Niskanen R. Fallahi E. (1998). Nitrogen and calcium nutrition and fruit quality of commercial apple cultivars grown in Finland. J. Plant Nutr. 21, 2389–2402. doi: 10.1080/01904169809365572

[B13] Falchi R. D’Agostin E. Mattiello A. Coronica L. Spinelli F. Costa G.. (2017). ABA regulation of calcium-related genes and bitter pit in apple. Postharvest Biol. Technol. 132, 1–6. doi: 10.1016/j.postharvbio.2017.05.017

[B14] Fazio G. Lordan L. Grusak M. Francescatto P. Robinson T. (2020). Mineral nutrient profiles and relationships of ‘Honeycrisp’ grown on a genetically diverse set of rootstocks under Western new York climatic conditions. Sci. Hortic. 266, 108477. doi: 10.1016/j.scienta.2019.05.004

[B15] Ferguson I. B. Watkins C. B. (1989). Bitter pit in apple fruit. Hortic. Rev. 11, 289–355. doi: 10.1002/9781118060841.ch8

[B16] Gavlak R. G. Horneck D. A. Miller R. O. (2005). Soil, plant and water reference methods for the Western region. 3rd ed (Western Coordinating Committee on Nutrient Management).

[B17] Goulao L. F. Oliveira C. M. (2008). Cell wall modifications during fruit ripening: when a fruit is not the fruit. Trends Food Sci. Technol. 19, 4–2. doi: 10.1016/j.tifs.2007.07.002

[B18] Hocking B. Tyerman S. D. Burton R. A. Gilliham M. (2016). Fruit calcium: Transport and physiology. Front. Plant Sci. 7, 569. doi: 10.3389/fpls.2016.00569 27200042 PMC4850500

[B19] Klein J. D. Hanzon J. Irwin P. L. Ben-Shalom N. Lurie S. (1995). Pectin esterase activity and pectin methyl esterification in heated ‘Golden delicious’ apples. Phytochemistry 39, 491–494. doi: 10.1016/0031-9422(94)00927-L

[B20] Lanauskas J. Kvikliene N. (2006). Effect of calcium foliar application on some fruit quality characteristics of ‘Sinap orlovskij’ apple. Agron. Res. 4, 31–36.

[B21] Lim C. White P. J. (2005). A cellular hypothesis for the induction of blossom-end rot in tomato fruit. Ann. Bot. 95, 571–581. doi: 10.1093/aob/mci065 15642726 PMC4246855

[B22] Liu Q. Talbot M. Llewellyn D. J. (2013). Pectin methylesterase and pectin remodelling differ in the fibre walls of two *gossypium* species with very different fibre properties. PLoS ONE 8 (6), e65131. doi: 10.1371/journal.pone.0065131 23755181 PMC3673955

[B23] Lordan J. Fazio G. Francescatto P. Robinson T. (2019). Horticultural performance of ‘Honeycrisp’ grown on a genetically diverse set of rootstocks under Western new York climatic conditions. Sci. Hortic. 257, 108686. doi: 10.1016/j.scienta.2019.108686

[B24] Marini R. P. Baugher T. A. Muehlbauer M. Sherif S. Crassweller R. Schupp J. R. (2020). Verification and modification of a model to predict bitter pit for ‘Honeycrisp’ apples. Hortscience 12, 1882–1887. doi: 10.21273/HORTSCI15301-20

[B25] Marini R. P. Lavely E. K. Baugher T. A. Crassweller R. Schupp J. R. (2022). Using logistic regression to predict the probability that individual ‘Honeycrisp’ apples will develop bitter pit. Hortscience 3, 391–399. doi: 10.21273/HORTSCI16081-21

[B26] Meyer G. A. Keliher P. N. (1992). “An overview of analysis by inductively coupled plasma-atomic emission spectrometry,” in Inductively coupled plasmas in analytical atomic spectrometry, vol. 2 . Eds. Montaser A. Golightly D. W. (New York, NY: VCH Publishers), 473–516.

[B27] Picchioni G. A. Watada A. E. Conway W. S. Whitaker B. D. Sams C. E. (1998). Postharvest calcium infiltration delays membrane lipid catabolism in apple fruit. J. Agric. Food. Chem. 46, 2452–2457. doi: 10.1021/jf971083e

[B28] Picchioni G. A. Watada A. E. Whitaker B. D. Reyes A. (1996). Calcium delays senescence-related membrane lipid changes and increases net synthesis of membrane lipid components in shredded carrots. Postharvest Biol. Technol. 9, 235–245. doi: 10.1016/S0925-5214(96)00051-8

[B29] Ralet M. C. Dronnet V. Buchholt H. C. Thibaulta J. F. (2001). Enzymatically and chemically de-esterified lime pectins: characterisation, polyelectrolyte behaviour and calcium binding properties. Carbohydr. Res. 336, 117–125. doi: 10.1016/S0008-6215(01)00248-8 11689182

[B30] Reid M. Kalcsits L. (2021). Water deficit timing affects physiological drought response, fruit size, and bitter pit development for ‘Honeycrisp’ apple. Plants 9, 874. doi: 10.3390/plants9070874 PMC7412486 32660084

[B31] Saure M. C. (2005). Calcium translocation to fleshy fruit: its mechanism and endogenous control. Sci. Hortic. 105, 65–89. doi: 10.1016/j.scienta.2004.10.003

[B32] Sherif S. M. (2022). Rootstock effects on tree growth and yield of ‘Honeycrisp’ apple under Virginia state climatic conditions (Virginia Cooperative Extension), SPES–398NP.

[B33] Torres E. Recasens I. Lordan J. Alegre S. (2017). Combination of strategies to supply calcium and reduce bitter pit in ‘Golden delicious’ apples. Sci. Hortic. 217, 179–188. doi: 10.1016/j.scienta.2017.01.028

[B34] Val J. Gracia M. A. Monge E. Blanco A. (2008). Visual detection of calcium by GBHA staining in bitter pit-affected apples. Food Sci. Technol. Int. 14, 315–319. doi: 10.1177/1082013208097191

[B35] Valverdi N. A. Kalcsits L. (2021). Rootstock affects scion nutrition and fruit quality during establishment and early production of ‘Honeycrisp’ apple. HortScience 56, 261–269. doi: 10.21273/HORTSCI15488-20

[B36] van den Hoogen B. M. van Weeren P. R. Lopes-Cardozo M. van Golde L. M. Barneveld A. van de Lest C. H. (1998). A microtiter plate assay for the determination of uronic acids. Anal. Biochem. 15, 107–111. doi: 10.1006/abio.1997.2538 9514779

[B37] White P. J. Broadley M. R. (2003). Calcium in plants. Ann. Bot. 92, 487–511. doi: 10.1093/aob/mcg164 12933363 PMC4243668

[B38] Yermiyahu U. Nir S. Ben-Hayyim G. Kafkafi U. (1994). Quantitative competition of calcium with sodium or magnesium for sorption sites on plasma membrane vesicles of melon (Cucumis melo l.) root cells. J. Membr. Biol. 138, 55–63. doi: 10.1007/BF00211069 8189432

