# Supplementary material for: Rootstock effects on bitter pit incidence in ‘Honeycrisp’ apples are associated with changes in fruit’s cell wall chemical properties
Source: Front Plant Sci. 2022 Oct 13;13:1034664. doi: 10.3389/fpls.2022.1034664 (PMC9606712; doi:10.3389/fpls.2022.1034664)
Supplement: Supplementary file 1 [file DataSheet_1.docx]

| **Nutrient** | **P**  **(ppm)** | **K**  **(ppm)** | **Ca**  **(ppm)** | **Mg**  **(ppm)** | **Zn**  **(ppm)** | **Mn**  **(ppm)** | **Cu**  **(ppm)** | **Fe**  **(ppm)** | **B**  **(ppm)** |
| --- | --- | --- | --- | --- | --- | --- | --- | --- | --- |
| **Concentration** | 5.24 | 61.21 | 1005 | 230.07 | 1.54 | 6.45 | 1.29 | 17.64 | 0.45 |

**Supplementary Table S1.** Nutrient analysis of the soil at the Saunders Brothers Orchard, Piney River, Virginia, United States (39 °06ʹ36.0 ʹN 78 °16ʹ48.0 ʹW).

**Supplementary Table S2.** Leaf nutrient coencetration (ppm) for ‘Honeycrisp’ apple trees grafted on ‘B.10’, ‘G.41’, and ‘V.6’ rootstocks.

| **Rootstock** | **N (ppm)** | **P (ppm)** | **K (ppm)** | **Ca (ppm)** | **Mg (ppm)** | **S (ppm)** | **Zn**  **(ppm)** | **Mn (ppm)** | **Fe**  **(ppm)** | **Cu (ppm)** | **B**  **(ppm)** |
| --- | --- | --- | --- | --- | --- | --- | --- | --- | --- | --- | --- |
| B.10 | 18200 | 1400 | 12100 | 16500 | 3100 | 1600 | 222.80 | 241.80 | 37.20 | 10.20 | 37.60 |
| G.41 | 17200 | 1300 | 13400 | 14000 | 2400 | 1700 | 311.00 | 172.40 | 30.80 | 11.40 | 40.80 |
| V.6 | 18000 | 1500 | 12400 | 12500 | 3100 | 1600 | 283.60 | 132.60 | 38.80 | 8.20 | 36.20 |

**Supplementary Table S3.** Effect of rootstock on the cell wall-bound Ca^2+^ (%) in ‘Honeycrisp’ apples.

| **Rootstock** | **Total Ca^2+^**  **(mmol kg^-1^ skin tissue)** | **Cell wall-bound Ca^2+^ (mmol kg^-1^ skin tissue)** | **Cell wall-bound Ca^2+^ (%)** |
| --- | --- | --- | --- |
| **B.10** | 19.77 | 14.89 | 75.08 |
| **G.41** | 12.03 | 9.92 | 82.97 |
| **V.6** | 11.83 | 10.05 | 84.82 |
